# Phylogenomic approaches reveal a robust time-scale phylogeny of the Terminal *Fusarium* Clade

**DOI:** 10.1186/s43008-024-00147-8

**Published:** 2024-06-07

**Authors:** Andrés Felipe Lizcano Salas, Jorge Duitama, Silvia Restrepo, Adriana Marcela Celis Ramírez

**Affiliations:** 1 Grupo de Investigación Celular y Molecular de Microorganismos Patógenos (CeMop), Universidad de los Andes, Bogotá, Colombia; 2 Systems and Computing Engineering Department, Universidad de los Andes, Bogotá, Colombia; 3 Chemical Engineering Department, Universidad de los Andes, Bogotá, Colombia

**Keywords:** *Bisifusarium*, Diversification time, Evolution, *Neocosmospora*, Neogene, Orthologs, *Rectifusarium*

## Abstract

The Terminal *Fusarium* Clade (TFC) is a group in the *Nectriaceae* family with agricultural and clinical relevance. In recent years, various phylogenies have been presented in the literature, showing disagreement in the topologies, but only a few studies have conducted analyses on the divergence time scale of the group. Therefore, the evolutionary history of this group is still being determined. This study aimed to understand the evolutionary history of the TFC from a phylogenomic perspective. To achieve this objective, we performed a phylogenomic analysis using the available genomes in GenBank and ran eight different pipelines. We presented a new robust topology of the TFC that differs at some nodes from previous studies. These new relationships allowed us to formulate new hypotheses about the evolutionary history of the TFC. We also inferred new divergence time estimates, which differ from those of previous studies due to topology discordances and taxon sampling. The results suggested an important diversification process in the Neogene period, likely associated with the diversification and predominance of terrestrial ecosystems by angiosperms. In conclusion, we presented a robust time-scale phylogeny that allowed us to formulate new hypotheses regarding the evolutionary history of the TFC.

**Supplementary Information:**

The online version contains supplementary material available at 10.1186/s43008-024-00147-8.

## Introduction

The “Terminal *Fusarium* Clade” (TFC) is a group in the *Nectriaceae* family (Hypocreales) that comprises species with agricultural and clinical relevance (van Diepeningen and de Hoog 2016; Sáenz et al. 2020; Geiser et al. 2021). It includes aggressive phytopathogens that can cause devastating diseases in cereals and other important crops, resulting in annual losses of billions of dollars for the agricultural sector worldwide (van Diepeningen and de Hoog 2016; Ekwomadu and Mwanza 2023; Han et al. 2023). Several species are well-known opportunistic human pathogens that are the major cause of fungal keratitis and nondermatophite mold onychomycosis, and this species pose a risk to immunocompromised patients for invasive and disseminated infections with high mortality despite antifungal therapy (Garnica and Nucci 2013; Sáenz et al. 2020; Brown et al. 2021; Nucci and Anaissie 2023). Historically, these infections have been called “fusariosis” (Garnica and Nucci 2013; van Diepeningen and de Hoog 2016). Recently, there have been reports of species in this group infecting animals such as sea turtles (Smyth et al. 2019; Gleason et al. 2020).

Gräfenhan et al. (2011) coined the term TFC in a study that reevaluated the “*Fusarium *sensu Wollenweber” concept (based on the morphological character of *Fusarium*-like conidia) by molecular phylogenetic analysis. This research splits this concept into two significant groups within the *Nectriaceae* family: the “Terminal *Fusarium* Clade”, which contains a group of anamorph and teleomorph genera centered around the *Giberella* clade, and the “Basal *Fusarium*-like clade”, which is divided into seven monophyletic genera (Gräfenhan et al. 2011). However, the backbone of this tree had low statistical support (Gräfenhan et al. 2011). Therefore, Schroers et al. (2011) conducted a phylogenetic analysis of the TFC using five molecular markers. As a result, these authors obtained a similar tree with a better backbone support of the TFC, showing that *Geejayessia*, a newly described genus, and *Cyanonectria* are distinct phylogenetic lineages and are not part of the *Fusarium s. str.* concept (this concept considers *Fusarium s. str.* to be characterized only by the *Gibberella* clade) (Schroers et al. 2011). Therefore, the TFC defines a monophyletic group of species with *Fusarium*-like conidia in the *Nectriaceae* family separated from a polyphyletic group with *Fusarium*-like conidia known as the “Basal *Fusarium*-like clade” (Gräfenhan et al. 2011; Schroers et al. 2011).

Since the arrival of the "one fungus = one name" movement (Taylor 2011), the taxonomy of the members of the TFC has been discussed and redefined at least twice. One of the first publications redefining the taxonomy of the TFC was made by O'Donnell et al. (2013), who proposed three representative nodes within the TFC: Node F1, representing the whole TFC group, which was selected in this study to define the genus *Fusarium*; Node F2, representing a second potential proposal for a monophyletic *Fusarium* genus definition with a higher boostrap support than the F1 node that excludes the *Fusarium ventricosum* species complex and *Fusarium dimerum* species complex; and Node F3, which represented the *Gibberella* clade. There are two main points of view in this disagreement: either the clade is composed of one genus named *Fusarium* (Geiser et al. 2013, 2021) or the clade is composed of multiple genera (Lombard et al. 2015; Sandoval-Denis and Crous 2018; Crous et al. 2021). The proposal to maintain a single genus named *Fusarium* is based on the monophyletic nature of the TFC, historical precedence, and practical considerations for the clinical management of infections (O’Donnell et al. 2020; Geiser et al. 2021; de Hoog et al. 2023). The division of the TFC into ten genera proposed by Crous et al. (2021) is based on phenotypical, biochemical (based on the production of secondary metabolites), and ecological data of the group. Based on this information and the lack of consensus in the mycology community, we will follow the nomenclature proposed by Crous et al. (2021) during this study due to its integrative approach.

**Fig. 1:**
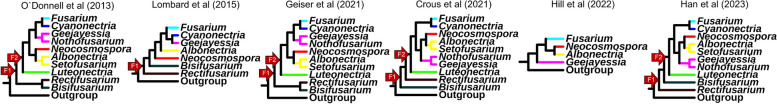
Comparison of topologies in previous studies using the new nomenclature. The F1 and F2 nodes from O’Donnell et al. (2013) are marked by arrows in each tree

The phylogenetic relationships of the TCF remain under active discussion. In recent years, different phylogenetic trees with disagreements in topology have been presented in the literature (Fig. 1). Most studies on phylogenetic relationships in the TFC have been carried out with a multi-locus approximation between 2 and 19 genes, leading to challenges in inferring the topology of the F1 node that represents all the genera included in the TFC because of the low support obtained (O’Donnell et al. 2013; Lombard et al. 2015; Crous et al. 2021; Geiser et al. 2021). In addition, this led to discordances in the relationships between the different genera (Fig. 1). A recent phylogenomic study published by Hill et al. (2022) included only four genera of the TFC. The first phylogenomic approach using all the genera was recently performed with excellent robustness of the F1 node but only used one approximation (maximum-likelihood approach using IQ-Tree) for the analysis (Han et al. 2023). Previous studies have shown that different approximations in a phylogenomic analysis could lead to varying tree topologies (Ametrano et al. 2019), even in the TFC (Hill et al. 2022). Hence, we considered that a study that uses multiple phylogenomic approximations to verify this newly proposed topology is still needed.

Understanding the time-scale of origin and diversification of fusarioid genera is crucial for reconstructing their evolutionary history. However, existing studies on the TFC's divergence times are limited. O'Donnell et al. (2013) and Hill et al. (2022) included all or a subset of genera, but their analyses were based on phylogenies with uncertain relationships. Inaccurate tree topologies can significantly distort divergence time estimates. Therefore, a time-scale phylogeny with a well-supported and robust topology for the entire TFC is necessary to obtain reliable estimates of origin and diversification times.

This project aims to understand the evolutionary history of the TFC from a phylogenomic perspective. The availability of genome assemblies in public databases presents an excellent opportunity to improve phylogenetic inference, aiding in defining the evolutionary history of this group of genera. To this end, 1) we inferred the TFC species tree from assemblies of eighty-one high-quality genomes of fusarioid species, and 2) we inferred the divergence time of the fusarioid genera that comprise the F1 node (Geiser et al. 2013).

## Materials and methods

### GenBank assemblies and gene prediction

Eighty-one genome assemblies of species in the TFC, two sister species (*Neonectria coccinea* and *Neonectria ditissima*), and an outgroup species (*Rugonectria rugulosa*) from GenBank (https://www.ncbi.nlm.nih.gov/genbank/) were used in this study (Additional file 2: Table S1). The genes were predicted through Braker2 v2.1.6 (Brůna et al. 2021) using the fungus option and OrthoBDv10 of *Hypocreales* (Kriventseva et al. 2018) as protein data input. The completeness of the predictions was verified by BUSCO v5.2.2 with the fungus lineage (Manni et al. 2021).

### Phylogenomic inference of species tree

OrthoFinder2 v2.5.4 (Emms and Kelly 2019) was run with default parameters from the predicted exonic sequence of genes with the two sister species *Ne. coccinea* and *Ne. ditissima* and the outgroup *Ru. rugulosa* to determine single-copy orthologs (SCOs). Then, the SCOs were aligned with two software programs: MAFFT v7.490 using the E-INS-I option and a maximum of ten iterations (Katoh and Standley 2013), and Muscle v5.1 with default parameters (Edgar 2004). The positions represented solely by gaps and unknown nucleotides (N) were removed.

For each set of alignments, coalescent-based trees were inferred. First, the gene trees were inferred using two programs: RAxML-NG v1.1 (Kozlov et al. 2019) using the MRE-based bootstrap convergence criterion with a maximum of 1000 replicates and IQ-Tree v2.2.03 with the 1000 UFboostrap (Hoang et al. 2018; Minh et al. 2020b). ModelFinder (Kalyaanamoorthy et al. 2017) and ModelTest-ng (Darriba et al. 2020) estimated the best fit evolutionary model for each SCO under the AICc criteria for IQ-Tree and RAxML-NG, respectively. Then, each set of gene trees was used as input from the software ASTRAL-III v5.7.8 to infer the species tree with the default parameters (Zhang et al. 2018). Finally, using the same alignments and the best fit evolutionary model previously obtained, a set of concatenated trees was inferred using IQ-Tree (Chernomor et al. 2016; Minh et al. 2020b) and RAxML-NG (Kozlov et al. 2019) with a partition model where each partition was an SCO. In total, eight trees were inferred and compared. For each tree, the genealogical concordance factor (gCF), site concordance factor (sCF) (Minh et al. 2020a), and local posterior probability (lpp) (Zhang et al. 2018) were used as local support values. The normalized quartet score (NQS) was used as a tree support value (Zhang et al. 2018).

A species tree of only the genus *Fusarium s. str.* was inferred as described above using the SCOs previously determined for the whole dataset to make a local inference. The outgroup for these trees was *Neocosmospora vasinfecta*. Finally, the selected species tree was compared to that obtained in the previous step of OrthoFinder (Emms and Kelly 2018, 2019).

### Divergence time tree inference

Initially, the SCOs were evaluated to define their “clock-like” behavior by calculating the degree of violation of a molecular clock (DVMC) (Liu et al. 2017) in PhyKIT v1.11.7 (Steenwyk et al. 2021) using the gene tree dataset obtained by MAFFT + RAxML-NG. Then, the 50 genes with lower DVMC were selected (Additional file 2: Table S2). The substitution rate of the 50 genes was estimated using baseml in the PAML package v4.9 (Yang 2007) with the GTR + G model. Finally, this substitution rate was used to calculate the gamma distribution shape and scale using the following formulas: shape = *(s/s)*^*2*^ and scale = *s/s*^*2*^, where *s* is the substitution rate (Steenwyk et al. 2019).

Eight approximations were performed to find the best set of prior parameters in the MCMCTree functionality (Yang and Rannala 2006) of the PAML package v4.9 (Yang 2007). The parameters evaluated were the substitution model (Sm), the clock model (Cm), and the gamma distribution of sigma2 (σ^2^). Two priors were defined for each parameter: JC and HKY + G4 for substitution models, the independent ratio (IR) and the autocorrelated ratio (AR) for the clock model, and G(1,4.5) and G(1,10) for the σ^2^ distribution. For all sets of parameters, the Hessian and gradient were measured using two points of node calibration: “ < 1.45” for the root and “ < 0.9 > 0.5” for the node of the most recent common ancestor (MRCA) between *Fusarium* and *Neocosmospora* (Lutzoni et al. 2018), the time scale is 100 million years (Myr). The divergence time for each set of parameters was inferred in two independent runs to verify convergence on MCMCTree with 30,000 generations, posterior sampling every ten generations, and a 10% burn-in.

We performed other simulations with selected priors (Sm = JC, Cm = IR, σ^2^ distribution of G(1,10)) to analyze the impact of the number of loci. Three sets of loci were used: the top 10, 50, and 100 genes with the lowest DVMC. The substitution rate, gradient and Hessian, and time divergence were inferred as previously described.

## Results

### A new species tree for the Terminal *Fusarium* Clade

**Fig. 2:**
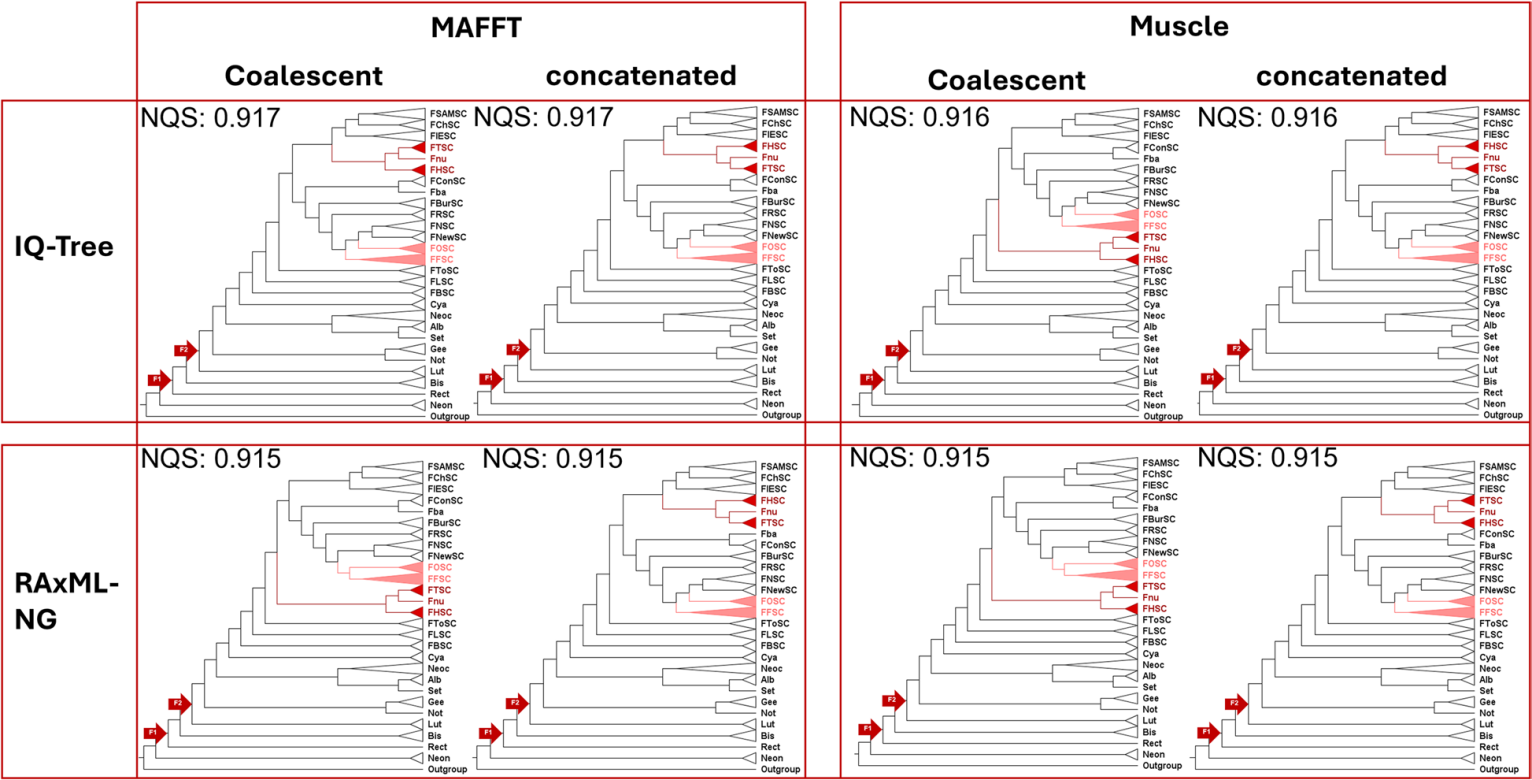
Comparison of collapsed species tree of the TFC with different pipelines. For the coalescent analysis, refer to ASTRAL. The F1 and F2 nodes from O’Donnell et al. (2013) are marked by arrows on each tree. The red and pink represent clades with disagreements between tree topologies. NQS = normalized quartet score, Alb = *Albonectria*, Bis = *Bisifusarium*, Cya = *Cyanonectria*, FBSC = *Fusarium buharicum* species complex, FLSC = *Fusarium lateritium* species complex, FToSC = *Fusarium torreayae* species complex, FFSC = *Fusarium fujikuroi* species complex, FOSC = *Fusarium oxysporum* species complex, FNewSC = *Fusarium newnesense* species complex, FNSC = *Fusarium nisikadoi* species complex, FRSC = *Fusarium redolens* species complex, FBurSC = *Fusarium burgessii* species complex, Fba = *Fusarium falsibabinda*, FConSC = *Fusarium concolor* species complex, FTSC = *Fusarium tricinctum* species complex, Fnu = *Fusarium nurragi*, FHSC = *Fusarium heterosporum* species complex, FIESC = *Fusarium incarnatum-equiseti* species complex, FChSC = *Fusarium chlamydosporum* species complex, FSAMSC = *Fusarium sambucinum* species complex, Gee = *Geejayessia*, Lut = *Luteonectria*, Neoc = *Neocosmospora*, Neon = *Neonectria*, Not = *Nothofusarium*, Rec = *rectifusarium*, Set = *Setofusarium*

An initial evaluation of the quality of the assemblies revealed a completeness of 93%—100% based on the conserved genes in fungi using BUSCO (Manni et al. 2021) (Additional file 2: Table S1). We integrated information from 1,049 single-copy orthologs (SCOs) across 81 TFC genome assemblies to generate a robust species tree inference. All trees exhibited high support for the inferred topology, with Normalized Quartet Score (NQS) between 0.915 and 0.917 (Fig. 2). Even with the increased support of all trees, two disagreements appeared in the genus *Fusarium*. First, the position of the clade composed by the *Fusarium heterosporum* species complex (FHSC), the *Fusarium tricinctum* species complex (FTSC), and *Fusarium nurragi* differ among some trees. The second discordance was the relationship between the *Fusarium fujikuroi* species complex (FFSC) and the *Fusarium oxysporum* species complex (FOSC). There were no discordances between trees in the relationships within other genera or species complexes (Fig. 2). Three topologies were observed with a predominance of a monophyletic relationship between FHSC, FTSC, *F. nurragi*, the *Fusarium sambucinum* species complex (FSamSC), the *Fusarium chlamydosporum* species complex (FChSC), and the *Fusarium incarnatum-equiseti* species complex (FIESC). This topology also determines a paraphyletic relation between FFSC and FOSC (Fig. 2).

Although a predominant topology was inferred, we preferred to make a local inference of the species tree for the genus *Fusarium s. str.*, using the same 1,049 SCOs and pipelines and *Neocosmospora vasinfecta* as an outgroup. This set of trees also had high support with NQS values between 0.914 and 0.915, and there were no differences between the topologies (Additional file 1: Fig. S1). Furthermore, this topology was consistent with the most frequently occurring topology resulting from analyses of all genera in the TFC (Fig. 2).

**Fig. 3:**
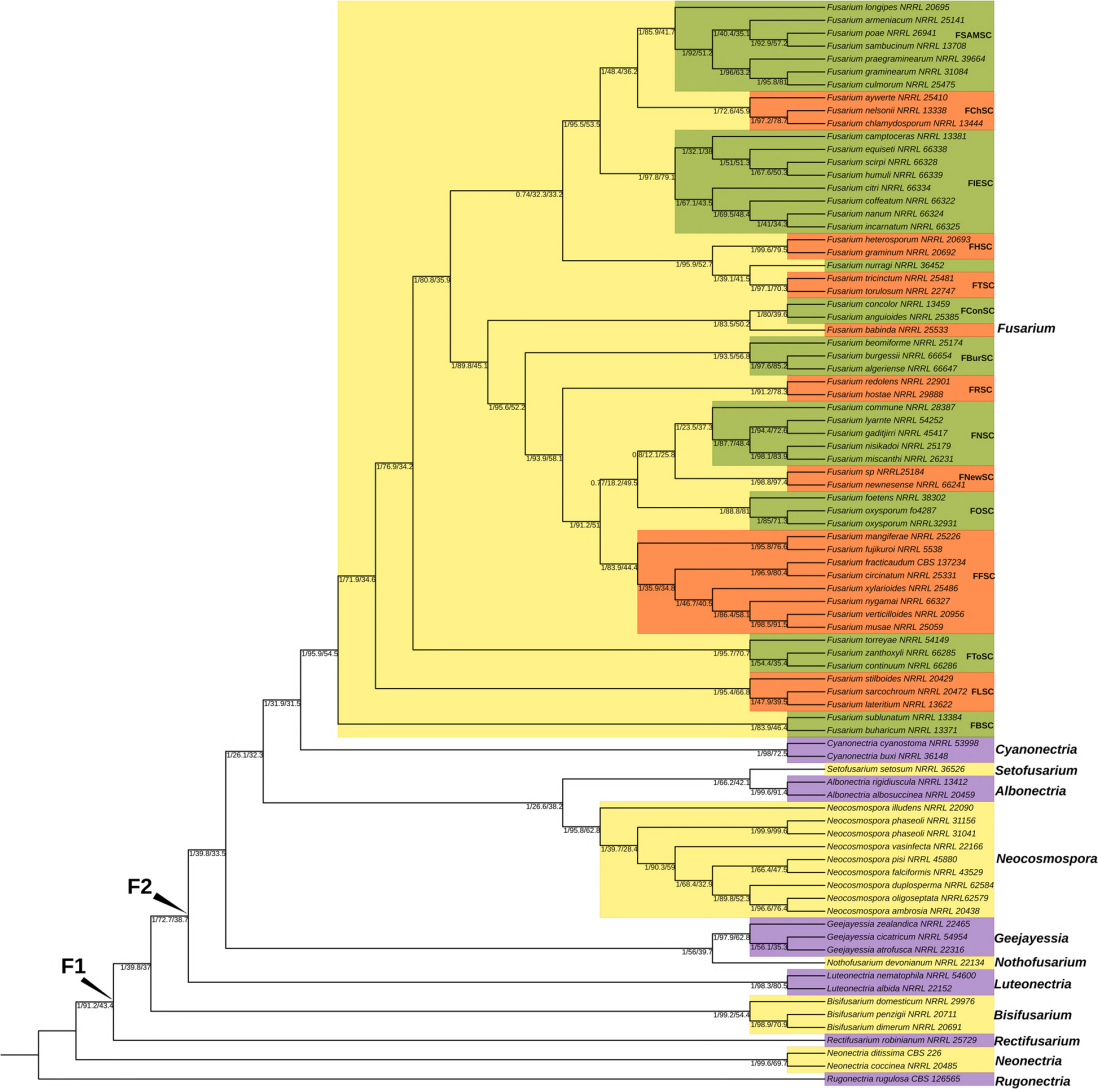
Cladogram of the selected TFC species tree. Partitioned maximum-likelihood analysis with 1,049 MAFFT-aligned SCOs performed in IQ-Tree. *Rugonectria rugulosa* was used as the outgroup. The F1 and F2 nodes from O’Donnell et al. (2013) are marked. Clades of *Neocosmospora* from O’Donnell (2000) and FIESC from Han et al. (2023) are highlighted. The Node values are “local posterior probability/general Concordance Factor/site Concordance Factor”. FBSC = *Fusarium buharicum* species complex, FLSC = *Fusarium lateritium* species complex, FToSC = *Fusarium torreayae* species complex, FFSC = *Fusarium fujikuroi* species complex, FOSC = *Fusarium oxysporum* species complex, FNewSC = *Fusarium newnesense* species complex, FNSC = *Fusarium nisikadoi* species complex, FRSC = *Fusarium redolens* species complex, FBurSC = *Fusarium burgessii* species complex, FConSC = *Fusarium concolor* species complex, FTSC = *Fusarium tricinctum* species complex, FHSC = *Fusarium heterosporum* species complex, FIESC = *Fusarium incarnatum-equiseti* species complex, FChSC = *Fusarium chlamydosporum* species complex, FSAMSC = *Fusarium sambucinum* species complex

The final tree that we propose in this study is shown in Fig. 3, which presented high general support with an NQS of 0.917. The support of the nodes varied between 0.74 and 1 local posterior probability (lpp), between 12.11 and 99.9 for the genealogical Concordance Factor (gCF), with a median of 87.7, and between 25.55 and 99.63 for the site Concordance Factor (sCF), with a median of 50.83. Node F1 validated the monophyletic relationship of the TFC with high support (1 lpp, 91.2 gCF, and 43.4 sCF) and determined that the first diverging genus was *Rectifusarium*. The node representing the monophyletic relation of each genus with more than one representative species in this study also exhibited high support (lpp 1, gCF > 90, and sCF > 70). Lower gCF values are observed in nodes representing relationships within the TFC genus or *Fusarium* species complexes, as well as relationships between species within a genus or species complex.

An analysis of the selected species tree compared to that generated by the previous step of Orthofinder revealed eight differences (Additional file 1: Fig. S2). These differences involved the relationships between genera in the F2 node; the positions of *N. illudens* and *F. commune*; the relationships among FOSC, FNSC, and FNewSC; and the positions of the clade composed by *F*. *nurragi*, FHSC, and FTSC.

### The Neogene geological period: a time for species diversification in *Fusarium* and *Neocosmospora*

The analyses converged in log-likelihood for all the parameter sets (Table 1). The σ^2^ distribution prior and substitution models had a minimal impact on age estimates (Additional file 1: Fig. S3). The clock model had the most significant impact on age estimates (Additional file 1: Fig. S3), with the IR model predicting divergence times younger than those of the AR model. These results suggest that the clock model in time-tree inference is the most important prior.

**Table 1:** Comparison of parameters from different models in MCMCTree runs

Model	Lnl		ESS^a^		Acceptance ratio^b^	
	Run 1	Run 2	Run 1	Run 2	Run 1	Run 2
Sm: HKY + G4, Cm: AR, σ^2^: G(1,10)	-4356,314	-4359,685	1035	1055	0.31—0.47	0.18—0.31
Sm: HKY + G4, Cm: AR, σ^2^: G(1,4.5)	-4357,191	-4338,473	1448	632	0.26—0.41	0.30—0.63
Sm: HKY + G4, Cm: IR, σ^2^: G(1,10)	-3457,39	-3456,565	14,913	16,441	0.24—0.45	0.29—0.31
Sm: HKY + G4, Cm: IR, σ^2^: G(1,4.5)	-3450,78	-3458,556	15,792	16,599	0.31—0.41	0.29—0.36
Sm: JC, Cm: AR, σ^2^: G(1,10)	-3996,319	-3997,065	1646	2339	0.30—0.39	0.16—0.41
Sm: JC, Cm: AR, σ^2^: G(1,4.5)	-3990,786	-3993,109	1792	1461	0.26—0.34	0.19—0.35
Sm: JC, Cm: IR, σ^2^: G(1,4.5)	-3283,436	-3285,387	18,615	19,007	0.26—0.38	0.25—0.35
Sm: JC, Cm: IR, σ^2^: G(1,4.5)	-3282,935	-3285,003	18,222	17,740	0.27—0.39	0.28—0.38

^a^ESS of the Lnl parameter, ^b^range of acceptance ratio for all parameters. *LnL* Log-likelihood, *Sm* Substitution model, *cm* Clock model, *ESS* Effective sample size

We evaluated the log-likelihood, effective sample size (ESS), and acceptance ratio (Table 1) to select the optimal priors for the clock model. Among these, the IR model was the most favorable choice, maximizing log-likelihood, achieving a higher ESS, and maintaining a suitable acceptance ratio (0.20–0.40). For the substitution model, we chose the JC model because it is faster, considering that it has fewer parameters. Additionally, we selected a G(1,10) σ^2^ distribution to provide a broader distribution of this parameter for further analysis.

We analyzed the effect of the number of SCOs on node age estimation. This analysis revealed that the credibility intervals overlapped in most nodes for all datasets (Additional file 1: Fig. S4). Additionally, age estimates for basal nodes were younger when ten loci were used than when 50 or 100 loci were used. This suggests that the number of SCOs can influence divergence time inference, particularly for basal nodes. Based on this observation, and the minimal difference observed between the trees generated using 50 and 100 SCOs (Additional file 1: Fig. S4), we selected the tree inferred using 100 loci for further analysis.

**Fig. 4:**
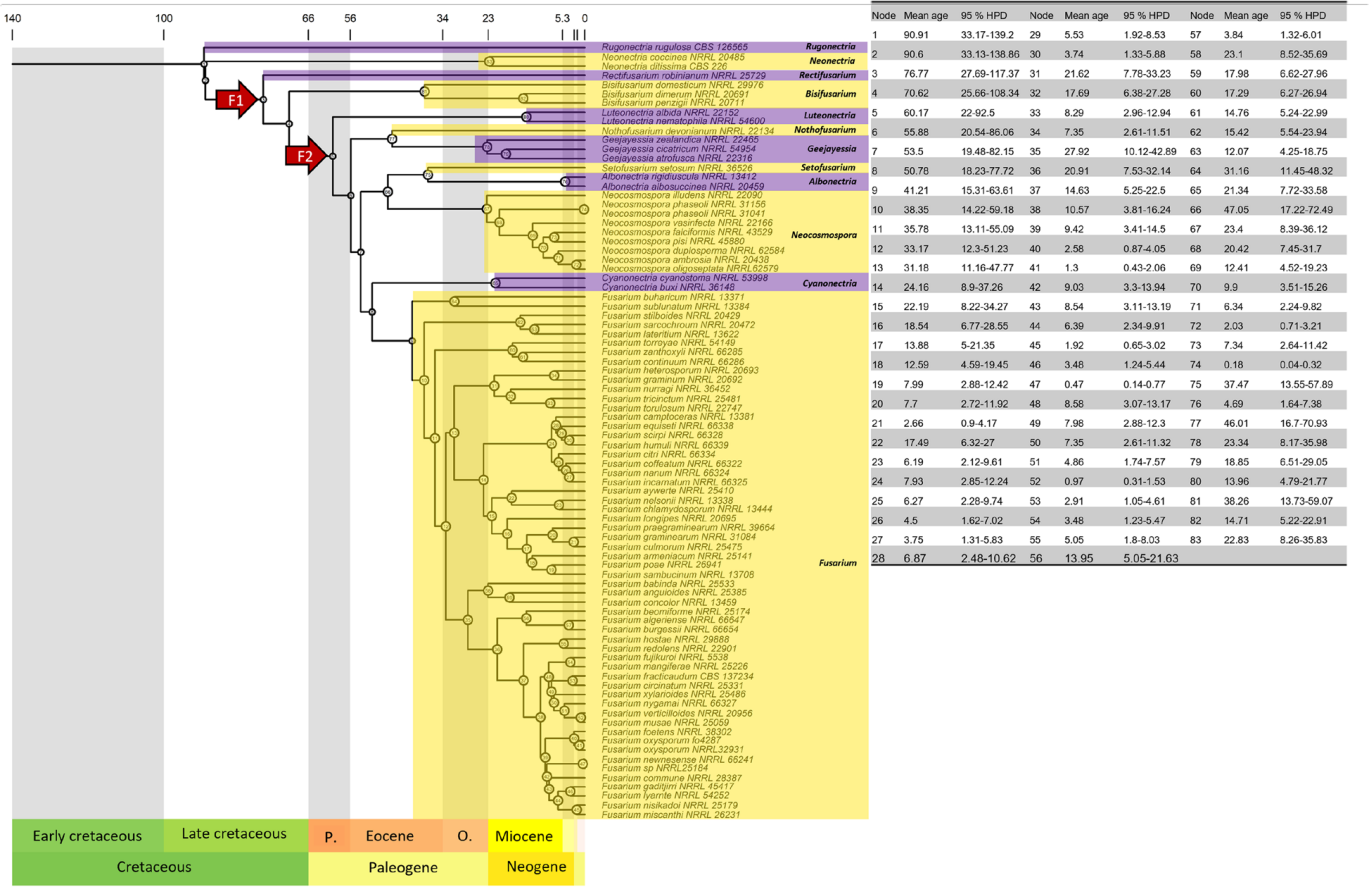
Time tree of the Terminal *Fusarium* Clade based on the JC substitution model, IR clock model, and σ^2^ distribution G(1,10) performed in MCMCTree. The values on top represent the time in Millions of years ago (Mya). The F1 and F2 nodes from O’Donnell et al. (2013) are marked by arrows. The mean age and highest probability density (HPD) of 95% for each node are shown in the table. P. = Paleocene, O. = Oligocene

The divergence time estimation tree indicated that the most likely crown age (the most recent common ancestor) of the TFC was in the Late Cretaceous, approximately 77 million years ago (Mya) (Fig. 4). The stem ages (divergence time from the ancestral lineage) of the clade containing the genera *Albonectria*, *Bisifusarium, Cyanonectria, Fusarium s. str.*, *Geejayessia, Luteonectria, Neocosmospora, Nothofusarium, Rectifusarium,* and *Setofusarium* were approximately 37, 51, 51, 46, 60, 47, 46, 77, and 37 Mya, respectively. These results suggest that the most likely origin of these fusarioid genera lies in the Paleogene and Neogene periods. Furthermore, we observed many speciation processes in *Fusarium* and *Neocosmospora* during the Neogene Period.

## Discussion

The phylogenomic approach has demonstrated its relevance for improving the inference of phylogenetic relationships in fungi (Ametrano et al. 2019; Steenwyk et al. 2019). Previous studies on phylogenetic relationships in the TFC were primarily conducted following a multi-locus approximation using a limited set of genes (O’Donnell et al. 2013; Lombard et al. 2015; Crous et al. 2021; Geiser et al. 2021). These limitations resulted in discordant tree topologies and low support for the critical F1 node. Here, we present the first comprehensive phylogenomic analysis of the TFC, employing multiple methods and encompassing significant sampling of species across genera.

Our results support the previously established monophyly of the TFC (O’Donnell et al. 2013; Geiser et al. 2021). Although monophyly is a crucial characteristic for defining a genus, it is not the sole criterion. This study did not aim to resolve the ongoing debate between single and multiple genera for the TFC (O’Donnell et al. 2013; Lombard et al. 2015; Sandoval-Denis and Crous 2018; Crous et al. 2021; Geiser et al. 2021). Instead, we provided a robust phylogenetic framework that can be used as the basis for unified TFC taxonomy. As highlighted in the literature, defining and redefining a genus requires an integrative approach that incorporates morphological and ecological data, as well as established monophyletic relationships (Chaverri et al. 2011; de Beer et al. 2014; Jones et al. 2014; Crous et al. 2021). Therefore, additional data are necessary to reach a consensus.

Comparing the obtained topology with those of previous studies, we noticed that the first diverging genus was *Rectifusarium*, as previously reported (Lombard et al. 2015; Han et al. 2023) in comparison with other studies that presented *Bisifusarium* (Crous et al. 2021) or a clade composed of *Rectifusarium* and *Bisifurarium* as the first to diverge (O’Donnell et al. 2013; Geiser et al. 2021). In contrast with other studies (O’Donnell et al. 2013; Crous et al. 2021; Geiser et al. 2021), we showed better support for the F1 node in gCF and lpp, which were 91.2 and 1, respectively. Additionally, the F1 node was supported by eight different analysis pipelines (Fig. 2). Together, these results show a well-supported topology for the F1 node (Fig. 3). For the F2 node, the relationships among the genera were the same as those obtained by Crous et al. (2021) and Han et al. (2023). In fact, we generally have an identical topology at the genus level as the study by Han et al. (2023).

For the genus *Neoscosmospora*, the previously described relationships among the clades proposed by O’Donnell (2000) were consistent with our reconstruction (Fig. 3). In *Fusarium*, the primary area of disagreement with past studies was the relationships among FFSC, FOSC, the *Fusarium newnesense* species complex (FNewSC), and the *Fusarium nisikadoi* species complex (FNSC) (Fig. 2; Additional file 1: Fig. S1) (O’Donnell et al. 2013; Crous et al. 2021; Geiser et al. 2021; Hill et al. 2022; Han et al. 2023). The topology proposed by Hill et al. (2022) is the most similar to our tree (Fig. 3). Our tree included FNewSC, which was absent from the study by Hill et al. (2022). This result revealed a monophyletic relationship between FNewSC and FNSC that was not described in previous studies (O’Donnell et al. 2013; Crous et al. 2021; Geiser et al. 2021; Han et al. 2023). The relationship between FNewSC and FNSC from this study differed from that presented in the previous phylogenomic analysis (Han et al. 2023). Also, our analysis revealed a discordance related to the position of the clade composed by FHSC, FTSC, and *F. nurragi.* However, we were unable to find this in the literature (O’Donnell et al. 2013; Crous et al. 2021; Geiser et al. 2021; Hill et al. 2022; Han et al. 2023). These findings show the relevance of using multiple approximations when studying a group with problematic phylogenetic relationships since other studies have also reported discordant relationships when performing multiple approximations (Ametrano et al. 2019; Hill et al. 2022).

Previous studies have shown disagreements about the phylogenetic relationships between *Fusarium camptoceras* and FIESC (Villani et al. 2019; Xia et al. 2019; Kim et al. 2020; Crous et al. 2021, 2022; Han et al. 2023). Some studies proposed *F. camptoceras* as an independent lineage, known as the *Fusarium camptoceras* species complex (FCamSC) (Xia et al. 2019; Crous et al. 2021, 2022), while others presented this species as a lineage related to the *Equiseti* clade in the FIESC, known as the *Camptoceras* clade (Villani et al. 2019; Kim et al. 2020; Han et al. 2023). Our results support the placement of *F. camptoceras* within the FIESC (gCF of 97.8 and lpp of 1) with a closer relationship to the *Equiseti* clade (Fig. 3). Species within the *Equiseti* clade are typically characterized by sporodochial macroconidia with elongated, whip-like apical cells (Xia et al. 2019). Interestingly, this characteristic is absent in basal species of the *Equiseti* clade, such as *Fusarium mucidum* and *Fusarium croceum* (Xia et al. 2019), and even in *F. camptoceras* (Marasas et al. 1998). This suggests that *F. camptoceras* is related to the basal species of the *Equiseti* clade, and these species probably belong to the *Camptoceras* clade. Unfortunately, basal species of the *Equiseti* clade could not be included because genomes are not available in public databases. Future works should include a wider range of species, including those described as part of the FCamSC (Xia et al. 2019), to understand the phylogenetic relationships between these species and the evolution of their sporodochial macroconidia characteristics.

The phylogenomic analysis revealed low support for the placement of *F. commune* within the FNSC (gCF = 23.5). Generally, species complexes with more than three members exhibited gCF values ranging from 72.6 to 97.5. This suggests that *F. commune* may belong to a distinct lineage closely related to the FNSC. Interestingly, if we consider *F. commune* to be separate from the FNSC, the gCF value supporting this species complex increases to 87.7, which falls within the expected range for established species complexes. These results align with previous studies that reported either low support for *F. commune* within the FNSC (Geiser et al. 2021; Han et al. 2023), or its placement entirely outside the FNSC (Laurence et al. 2016; Husna et al. 2021). In favor of considering this latter issue is the production of microconidia in chains and larger macroconidia in FNSC (Nirenberg 1997; Gams et al. 1999; Phan et al. 2004; Walsh et al. 2010; Wang et al. 2022), and the absence of these characteristics in *F. commune* (Skovgaard et al. 2003).

Additionally, the analysis of divergence times within the TFC revealed younger average ages for major nodes compared to previous studies (O’Donnell et al. 2013; Hill et al. 2022). While most of the comparable estimated ages overlap with the confidence intervals of past studies, these results offer a potentially more robust TFC diversification history due to two key strengths. First, this analysis is informed by a well-supported phylogenetic topology. For example, the phylogenomic tree (Fig. 4) exhibits different points of divergence compared to O’Donnell et al. (2013), such as the placement of *Bisifusarium* and *Rectifusarium*, the *Geejayessia*/*NothoFusarium* clade, and the *Neocosmospora*/*Albonectria*/*Setofusarium* clade. Second, we either incorporated a broader range of taxa or reported a greater number of estimated ages than previous studies. More specifically, we reported 80 node ages within the TFC, compared to 60 nodes in Hill et al. (2022) and only 19 nodes reported by O’Donnell et al. (2013).

The divergence time estimates provide valuable insights into the evolutionary history of the TFC. The analysis suggested a significant diversification of *Fusarium* and *Neocosmospora* species within the Neogene era (Fig. 4). This geological period coincided with several key events that potentially influenced fungal evolution, including relevant tectonic movements (such as the closure of the Panama Isthmus), climate change, and rapid diversification and terrestrial dominance of angiosperms that began in the Late Cretaceous (Rull 2011; Ramírez-Barahona et al. 2020; Benton et al. 2022). Notably, many *Fusarium* and *Neocosmospora* species interact closely with plants as pathogens, endophytes, or decomposers (van Diepeningen and de Hoog 2016; Sandoval-Denis et al. 2019; Hill et al. 2022). This ecological connection aligns with the established link between fungal and plant diversification (Lutzoni et al. 2018), leading us to propose the hypothesis that the close relationship between *Fusarium* and *Neocosmospora*, and plants may have driven speciation events of these fungal genera within the Neogene. Further studies are necessary to validate this hypothesis.

## Conclusion

In conclusion, we present a robust time-scale phylogeny that differs from those of previous studies on topology and divergence time. Phylogenomic approaches should be preferred when studying the evolutionary history of the TFC. Also, these results demonstrated the relevance of using multiple approximations in a phylogenomic study since a single approximation could show discordant results from the most likely topology. We expect that these results could help to define a unified taxonomy in the ongoing debate. Finally, these results allowed us to infer new hypotheses about the evolutionary history of the TFC that should be verified in future studies.

### Supplementary Information

Additional file 1: Fig. S1. Comparison of collapsed species tree of *Fusarium* with different pipelines. Red and pink represent the previous clades with disagreements. NQS = normalized quartet score, FBSC = *Fusarium buharicum* species complex, FLSC = *Fusarium lateritium* species complex, FToSC = *Fusarium torreayae* species complex, FFSC = *Fusarium fujikuroi* species complex, FOSC = *Fusarium oxysporum* species complex, FNewSC = *Fusarium newnesense* species complex, FNSC = *Fusarium nisikadoi* species complex, FRSC = *Fusarium redolens* species complex, FBurSC = *Fusarium burgessii* species complex, Ffa = *Fusarium falsibabinda*, FConSC = *Fusarium concolor* species complex, FTSC = *Fusarium tricinctum* species complex, Fnu = *Fusarium nurragi*, FHSC = *Fusarium heterosporum* species complex, FIESC = *Fusarium incarnatum-equiseti* species complex, FChSC = *Fusarium chlamydosporum* species complex, FSAMSC = *Fusarium sambucinum* species complex. Fig. S2. Comparison between selected species tree (left) and Orthofinder output tree based on STAG (right). The red lines show the relationships between trees, and the dots represent the nodes with discordances. Fig. S3. Correlation of node ages between different prior parameters. The scale in the “x” and “y” axis is 100 Myr, and the dashed line represents equality between age estimates. A-D) Comparison of the σ^2^ prior, E-H) comparison of the substitution model prior, and I-L) comparison of the clock model prior. Fig. S4.  Comparison of node ages between different numbers of loci. Dots represent the divergence time estimates, and lines represent the 95% credibility interval.  

Additional file 2: Table S1. Genomes from GenBank used in this study. Table S2. Statistics of single-copy ortholog (SCO) gene trees of the Terminal Fusarium Clade.

## Data Availability

All alignments produced in this study with the corresponding gene and species tree in the NEXUS format are available in the TreeBase database (https://www.treebase.org/treebase-web/home.html) with a study ID of 30374. The information about the dataset, alignment and tree statistics supporting the conclusions of this article are included within the article and its additional file.

## Abbreviations

Alb: *Albonectria*
AR: Autocorrelated ratio
Bis: *Bisifusarium*
Cm: Clock model
Cya: *Cyanonectria*
DVMC: Degree of violation of a molecular clock
ESS: Effective sample size
FBSC: *Fusarium buharicum* species complex
FBurSC: *Fusarium burgessii* species complex
FChSC: *Fusarium chlamydosporum* species complex
FConSC: *Fusarium concolor* species complex
FFSC: *Fusarium fujikuroi* species complex
FHSC: *Fusarium heterosporum* species complex
FIESC: *Fusarium incarnatum-equiseti* species complex
FLSC: *Fusarium lateritium* species complex
FNewSC: *Fusarium newnesense* species complex
FNSC: *Fusarium nisikadoi* species complex
FSAMSC: *Fusarium sambucinum* species complex
FOSC: *Fusarium oxysporum* species complex
FRSC: *Fusarium redolens* species complex
FToSC: *Fusarium torreayae* species complex
FTSC: *Fusarium tricinctum* species complex
gCF: Genealogical Concordance Factor
Gee: *Geejayessia*
HKY: Hasegawa-Kishino-Yano model
HPD: Highest Probability Density
IR: Independent Ratio
JC: Jukes-Cantor model
LnL: Log-likelihood
lpp: Local posterior probability
Lut: *Luteonectria*
MRCA: Most recent common ancestor
Mya: Million years ago
Myr: Million years
Neoc: *Neocosmospora*
Neon: *Neonectria*
Not: *Nothofusarium*
NQS: Normalized Quartet Score
Rec: *Rectifusarium*
sCF: Site Concordance Factor
SCOs: Single-Copy Orthologogs
Set: *Setofusarium*
Sm: Substitution model
s. str.: *sensu stricto*
TFC: Terminal *Fusarium* Clade
