# Supplementary material for: Phylogenomic approaches reveal a robust time-scale phylogeny of the Terminal Fusarium Clade
Source: IMA Fungus. 2024 Jun 7;15:13. doi: 10.1186/s43008-024-00147-8 (PMC11161934; doi:10.1186/s43008-024-00147-8)
Supplement: Supplementary file 1 — Additional file 1: Fig. S1. Comparison of collapsed species tree of Fusarium with different pipelines. Red and pink represent the previous clades with disagreements. NQS = normalized quartet score, FBSC = Fusarium buharicum species complex, FLSC = Fusarium lateritium species complex, FToSC = Fusarium torreayae species complex, FFSC = Fusarium fujikuroi species complex, FOSC = Fusarium oxysporum species complex, FNewSC = Fusarium newnesense species complex, FNSC = Fusarium nisikadoi species complex, FRSC = Fusarium redolens species complex, FBurSC = Fusarium burgessii species complex, Ffa = Fusarium falsibabinda, FConSC = Fusarium concolor species complex, FTSC = Fusarium tricinctum species complex, Fnu = Fusarium nurragi, FHSC = Fusarium heterosporum species complex, FIESC = Fusarium incarnatum-equiseti species complex, FChSC = Fusarium chlamydosporum species complex, FSAMSC = Fusarium sambucinum species complex. Fig. S2. Comparison between selected species tree (left) and Orthofinder output tree based on STAG (right). The red lines show the relationships between trees, and the dots represent the nodes with discordances. Fig. S3. Correlation of node ages between different prior parameters. The scale in the “x” and “y” axis is 100 Myr, and the dashed line represents equality between age estimates. A-D) Comparison of the σ2 prior, E-H) comparison of the substitution model prior, and I-L) comparison of the clock model prior. Fig. S4. Comparison of node ages between different numbers of loci. Dots represent the divergence time estimates, and lines represent the 95% credibility interval. [file 43008_2024_147_MOESM1_ESM.docx]

**Supplementary Figures**


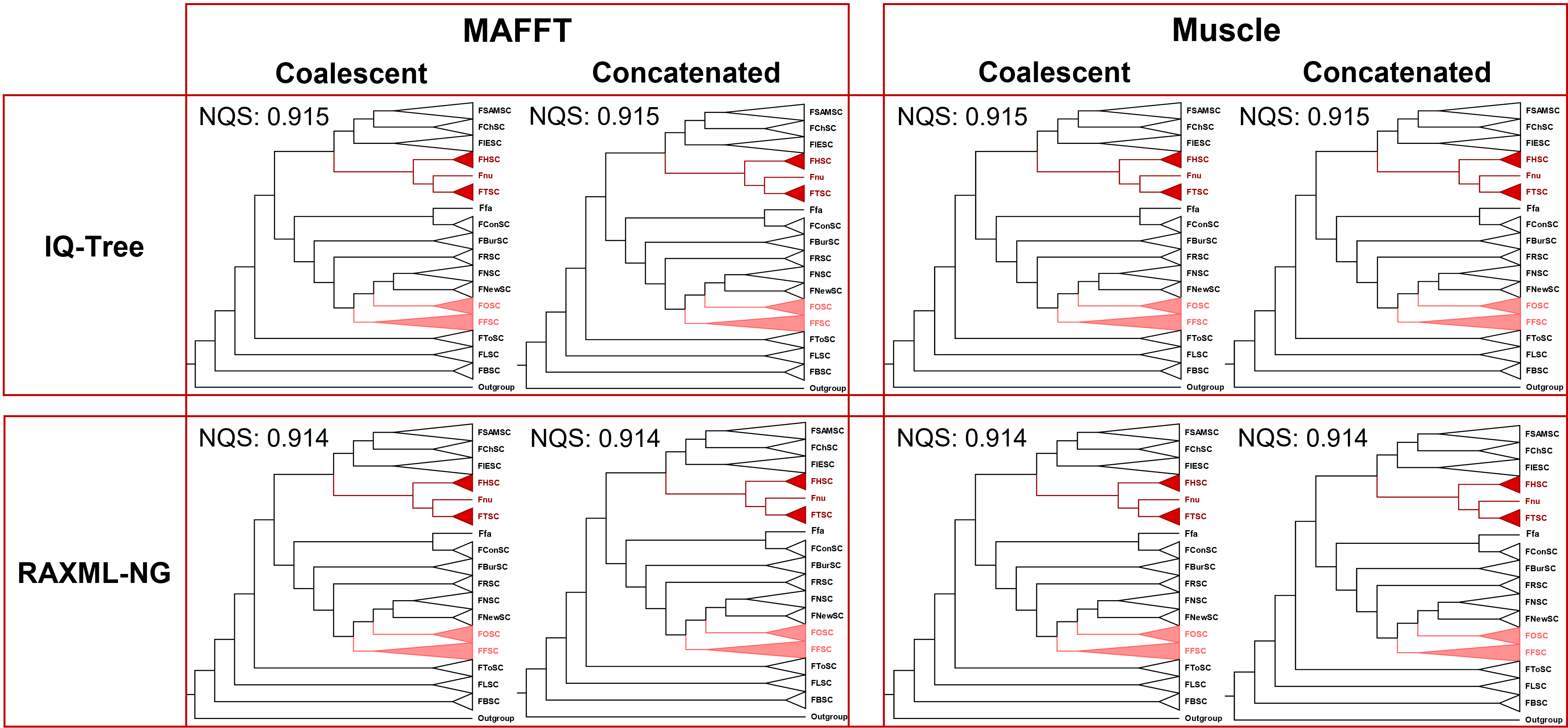


**Fig. S1**. Comparison of collapsed species tree of *Fusarium* with different pipelines. For the coalescent analysis, refer to ASTRAL. Red and pink represent the previous clades with disagreements. NQS = normalized quartet score, FBSC = *Fusarium buharicum* species complex, FLSC = *Fusarium lateritium* species complex, FToSC = *Fusarium torreayae* species complex, FFSC = *Fusarium fujikuroi* species complex, FOSC = *Fusarium oxysporum* species complex, FNewSC = *Fusarium newnesense* species complex, FNSC = *Fusarium nisikadoi* species complex, FRSC = *Fusarium redolens* species complex, FBurSC = *Fusarium burgessii* species complex, Ffa = *Fusarium falsibabinda*, FConSC = *Fusarium concolor* species complex, FTSC = *Fusarium tricinctum* species complex, Fnu = *Fusarium nurragi*, FHSC = *Fusarium heterosporum* species complex, FIESC = *Fusarium incarnatum-equiseti* species complex, FChSC = *Fusarium chlamydosporum* species complex, FSAMSC = *Fusarium sambucinum* species complex.


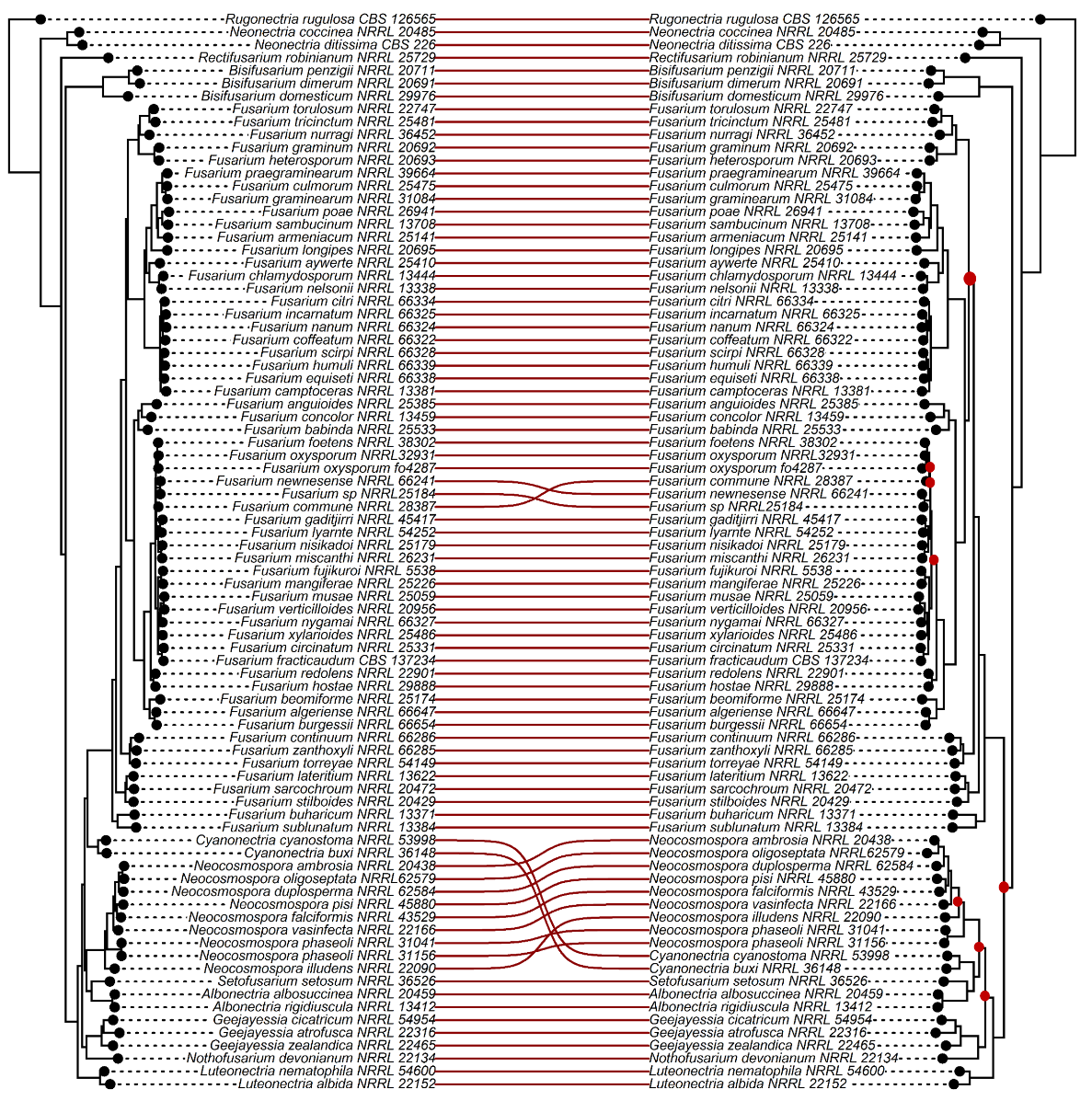


**Fig. S2**. Comparison between selected species tree (left) and Orthofinder output tree based on STAG (right). The red lines show the relationships between trees, and the dots represent the nodes with discordances.





**Fig. S3**. Correlation of node ages between different prior parameters in MCMCTree. The scale in the “x” and “y” axis is 100 millions years ago (Mya), and the dashed line represents equality between age estimates. **A-D)** Comparison of the σ^2^ prior, **E-H)** comparison of the substitution model prior, and **I-L)** comparison of the clock model prior.





**Fig. S4.** Comparison of node ages between using different numbers of SCOs based on the JC substitution model, IR clock model, and σ^2^ distribution G(1,10) performed in MCMCTree. Dots represent the divergence time estimates, and lines represent the 95% credibility interval.
